# The evolving landscape of minimally invasive procedures in musculoskeletal diseases—Part II

**DOI:** 10.3389/fmed.2026.1829343

**Published:** 2026-06-29

**Authors:** Fernando Saraiva, Esperanza Naredo

**Affiliations:** 1 Department of Rheumatology and Metabolic Bone Diseases, Centro Académico de Medicina de Lisboa, Unidade Local de Saúde de Santa Maria, Lisbon, Portugal; 2 Department of Rheumatology and Bone and Joint Research Unit, Hospital Universitário Jiménez Diaz, IIS-FJD, and Universidad Autónoma de Madrid, Madrid, Spain

**Keywords:** ultrasound guidance, minimally invasive musculoskeletal procedures, nerve blocks, ozonetherapy, platelet-rich plasma, prolotherapy, radiosynovectomy, thermal ablation

## Abstract

This review aims to cover a wide array of minimally invasive musculoskeletal procedures developed to address several articular and periarticular conditions locally. In part 2, we will cover nerve blocks, ozonetherapy, platelet-rich plasma and derivatives, prolotherapy, radiopharmaceuticals, sclerotherapy, thermal ablation, and intratissue percutaneous electrolysis. A review of guidelines covering these issues will also be presented. In part 1, in addition to details related to ultrasound guidance, we reviewed barbotage, botulinum toxin, corticosteroids, dry needling, gene therapy, hyaluronic acid, hydrodissection, and mesenchymal stem cells.

## Introduction

Rheumatic musculoskeletal diseases (RMD) present significant and persistent challenges to healthcare providers. These conditions can profoundly affect patients’ wellbeing, functional capacity, and overall quality of life. Furthermore, RMDs often impose a considerable burden on families and society at large, with wide-reaching socio-economic implications and potential consequences for mental health.

There is a growing array of local techniques, specifically minimally invasive musculoskeletal procedures (MIMSP), most of them being ultrasound-guided, developed to address articular and soft tissue manifestations of rheumatic diseases. These interventions are expanding the therapeutic landscape while simultaneously challenging the existing knowledge and skill set of rheumatologists.

The administration of drugs or autologous products directly into articular or soft tissue sites offers distinct advantages compared to systemic delivery. These include reduced systemic exposure and associated adverse effects, enhanced local bioavailability and, in some cases, lower overall costs. Despite these benefits, the effectiveness of certain intra-articular and soft tissue therapies remains a subject of debate. This uncertainty stems from limited data, methodological shortcomings, study heterogeneity, and a scarcity of high-quality evidence. Additionally, outcomes as reported by patients, such as pain relief and reduction in stiffness, are particularly susceptible to placebo effects, a phenomenon that is especially prominent with injectable treatments.

The primary objective of this research topic is to examine the underlying mechanisms of action, as well as the therapeutic efficacy and safety of MIMSP, covering a diverse range of interventions designed to address musculoskeletal manifestations.

Conducting rigorous investigations into these therapies is essential, particularly as the demand for effective management strategies continues to rise. Such approaches must be tailored to the unique needs of individual patients, ensuring that treatment remains both patient-centered and clinically relevant.

## Methods

A literature search was conducted using PubMed and Google Scholar as web search engines. Included key words were “minimally invasive musculoskeletal procedures,” “nerve blocks” AND “musculoskeletal,” “ozone therapy” AND “musculoskeletal,” “platelet-rich plasma” AND “musculoskeletal,” “prolotherapy” AND “musculoskeletal,” “radiopharmaceuticals” AND “musculoskeletal,” “radiosynovectomy” AND “musculoskeletal,” “sclerotherapy” AND “musculoskeletal,” “thermal ablation” AND “musculoskeletal,” “radiofrequency” AND “musculoskeletal,” and “intratissue percutaneous electrolysis.” No a priori exclusions were made concerning study design, publication date, or language. The only inscribed restriction was that the search should be limited to human studies. The search produced 2,385 results, of which 2,207 were excluded due to duplications, non-human studies, procedures for non-musculoskeletal applications, or for tumor pathologies. Finally, 178 publications were analyzed, aiming to retrieve information that could be useful for this review.

## The procedures

### Nerve blocks

A nerve block (NB) is a technique that is directed to a specific nerve or group of nerves, aiming to interrupt or reduce pain signals. There are several types of NBs according to the nature of the target: peripheral NBs, central NBs, and sympathetic NBs. NBs may be performed using chemical or physical agents. In the first group, we include local anesthetics (LA) and neurolytic substances as effector agents. In the second group, we include thermal ablation and percutaneous electric nerve stimulation (Table 1).

**TABLE 1:** Nerve blocks (NBs).

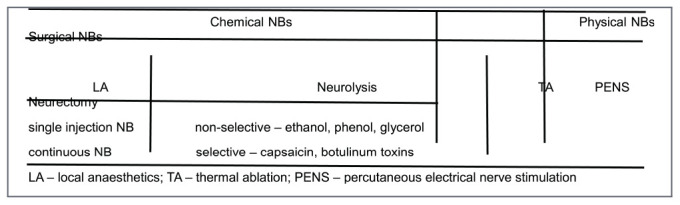

Neurolysis refers to the deliberate destruction of neural tissue to block nerve signals, aiming to treat chronic pain or spasticity. It may be temporary or permanent. Neurolysis is usually only considered when other pain management approaches, including other less invasive NBs, have failed. Chemical neurolysis or chemodenervation may be conducted using nonselective agents like ethanol, phenol, or glycerol, or through selective products like capsaicin and botulinum toxins. Neurolysis may also be achieved through thermal ablation or surgically through a neurectomy (cutting or removing a nerve surgically) (Table 1). However, the term neurolysis is also used to describe the surgical release of an entrapped nerve.

Percutaneous electric nerve stimulation is a technique consisting of the administration of an electric current to a nerve, aiming to decrease pain signals, a principle also known as neuromodulation. This is to be distinguished from nerve stimulation devices used to elicit a motor response, aiming to localize a nerve for NB.

We will focus on peripheral NBs, whereas thermal ablation and botulinum toxin are covered elsewhere in this review.

Common indications for NB include anesthesia in the context of trauma or perioperative pain management and analgesia for failed peripheral joints, neuropathic pain, and cancer pain. A NB can also be conducted for diagnostic purposes when used to identify the source of pain.

Nerve blocks can be performed using anatomical landmarks, nerve stimulation devices, or may be guided by fluoroscopy or ultrasonography, aiming to localize target nerves. Fluoroscopy-guidance still has its place in selected cases in the neuroaxis, but not in the appendicular skeleton, where ultrasound-guided (USG) NBs are the preferred method due to lack of radiation and increased sensitivity for deploying soft tissue components, like vessels and nerves.

Comparatively to anatomical landmarks or peripheral nerve stimulation guidance, USG NBs have demonstrated overall improved block safety and effectiveness, namely in terms of pain during the procedure, number of vascular punctures, shorter onset time, reduced procedure time, duration of adequate analgesia/anesthesia, lower local anesthetic dose, reduced complications incidence (including nerve injury and LA systemic toxicity), need for rescue analgesia through anesthesia, and overall block success rate (1–12). Moreover, the accepted threshold of electric nerve stimulation (0.5 mA) may be insufficient to trigger a muscle contraction in 25 % of patients, resulting in potential intraneural injection (13). However, deep-seated nerves may benefit from the concomitant use of electric nerve stimulation and US guidance, for confirmation of need tip placement, a method also known as “dual guidance” or “protective nerve stimulation” (4).

#### Local anesthetic nerve blocks

The most common form of NB uses a LA. LAs act by blocking Na-specific ion channels on neuronal cell membranes, inhibiting signal conduction. LA NBs are performed by injecting an anesthetic in the perineural space of the sensory nerve that innervates the area and proximal to it, providing anesthesia/analgesia distant to the block spot (14).

LAs may be differentiated according to drug class (amide, ester), concentration, onset time, maximum recommended dose, and effect duration. However, effect duration may vary depending on the injection site, technique, and patient response. In clinical practice, 2–20 mL of a long-lasting LA, like bupivacaine or ropivacaine, is usually preferred (Table 2).

**TABLE 2:** Local anesthetics—key properties.

Local anesthetic	Onset	Duration (no epinephrine)	Duration (with epinephrine)	Max dose (no Epi)	Max dose (with epi)
Lidocaine	Fast	30–60 min	60–240 min	4.5 mg/kg (max 300 mg)	7 mg/kg (max 500 mg)
Mepivacaine	Fast	60–180 min	120–360 min	5 mg/kg (max 400 mg)	7 mg/kg (max 550 mg)
Prilocaine	Intermediate	60–90 min	90–360 min	6 mg/kg (max 400 mg)	8 mg/kg (max 600 mg)
Bupivacaine	Slow	120–240 min	180–420 min	2.5 mg/kg (max 175 mg)	3 mg/kg (max 225 mg)
Levobupivacaine	Intermediate	180–360 min	240–480 min	2 mg/kg (max 150 mg)	3 mg/kg (max 225 mg)
Ropivacaine	Intermediate	120–360 min	180–420 min	3 mg/kg (max 200 mg)	3.5 mg/kg (max 250 mg)
Procaine	Slow	30–60 min	45–90 min	8 mg/kg (max 600 mg)	10 mg/kg (max 800 mg)
Chloroprocaine	Fast	20–30 min	30–90 min	11 mg/kg (max 800 mg)	14 mg/kg (max 1,000 mg)
Tetracaine	Slow	120–180 min	180–600 min	1.5 mg/kg (max 100 mg)	2.5 mg/kg (max 150 mg)

LAs can also be mixed (usually one LA with a rapid onset, with a LA with a longer effect), or epinephrine (a vasoconstrictor) may be added to allow higher doses of the LA (by slowing systemic absorption), to prolong its effect, or when bleeding is anticipated.

Multiple RCTs demonstrated that peripheral NBs significantly improve analgesia and reduce opioid needs across several joint and trauma surgeries compared to opioids alone, leading to a reduced incidence of side effects, cumulative opioid requirement, and earlier patient discharge. This also explains why NBs are becoming the gold standard in perioperative pain management (15, 16).

To perform anesthesia for surgical procedures or perioperative pain management, a higher concentration or volume of a LA is needed, compared to the amount used for analgesia in other situations of acute or chronic pain, and quite often NBs for surgeries use non-motor sparing NBs, which will not be covered in this review.

Two types of peripheral NB with LA can be conducted for pain control: single injection and continuous NB. In the last modality, an indwelling catheter is inserted percutaneously, usually with US guidance and connected to a continuous infusion pump for longer administration periods, because a major limitation of LA NB is its limited action length. However, there are some issues related to continuous NB, including catheter-associated infection and dislodgements. Nevertheless, there is no difference in pain reduction scores between the two methods, and continuous NB is beyond the scope of this review (15).

Non-surgical indications for LA NBs in the musculoskeletal system aim for pure sensory analgesia, that is, motor-sparing interventions that allow faster recovery times and earlier autonomous ambulation. Tables 3, 4 summarize the motor-sparing NBs and their primary sensory targets for each upper and lower limb joint, respectively. Technical details on these blocks are covered elsewhere (17–20).^1^,^2^, ^3^

**TABLE 3:** Motor-sparing analgesic blocks by upper-limb joint.

Joint	Primary sensory targets	Motor-sparing blocks	Notes
Shoulder (GH + AC)	Suprascapular (70%), Axillary (30%)	Suprascapular nerve block + Axillary nerve block ± Superior trunk block (low-volume)	Best combination for shoulder analgesia without phrenic palsy. Superior trunk adds coverage with minimal motor impact.
Elbow (HU, HR, PRU)	LABC, MABC, Superficial radial, sensory branches of median/ulnar	LABC block + MABC block + Superficial radial nerve block ± Low-volume median or ulnar sensory-only blocks	Provides circumferential elbow analgesia while preserving biceps, triceps, and forearm flexor/extensor strength.
Wrist (RC + Midcarpal)/hand	Median sensory, Ulnar sensory, superficial radial	Median nerve block at wrist + Ulnar nerve block at wrist + Superficial radial nerve block	Excellent for analgesia. Wrist blocks cover the entire hand without significant motor loss.
Thumb (CMC, MCP, IP)	Median (volar), superficial radial (dorsal), digital nerves	Superficial radial nerve block + Median nerve block + Thumb digital block	Ideal for CMC arthritis, trauma, or postoperative analgesia. Digital block is the most focused option.
Fingers (digital joints)	Proper digital nerves (paired plantar + dorsal)	Digital nerve blocks	Perfect for finger joints synovial biopsies. Digital blocks are the most targeted and fully motor-sparing.

GH, glenohumeral joint; AC, acromioclavicular joint; HU, humeroulnar joint; HR, humeroradial joint; PRU, proximal radiolunar joint; LABC, lateral antebrachial cutaneous nerve; MABC, medial antebrachial cutaneous nerve; RC, radiocarpal joint; CMC, carpometacarpal joint; IP, interphalangeal joint.

**TABLE 4:** Motor-sparing analgesic blocks by lower-limb joint.

Joint	Primary sensory targets	Motor-sparing blocks	Notes
Hip (Coxofemoral)	Femoral articular branches, Obturator articular branch, Accessory obturator, LFCN	PENG block + Supra-inguinal fascia iliaca (low volume) + LFCN ± Obturator articular branch block	PENG is the most motor-sparing option for the hip. FICB adds broader plexus coverage with minimal quadriceps weakness.
Knee (Tibiofemoral + Patellofemoral)	Saphenous nerve, nerve to vastus medialis, genicular branches, posterior capsule sensory branches	ACB + IPACK block ± Genicular nerve blocks (SM, SL, IM)	ACB preserves quadriceps strength. IPACK provides posterior capsule analgesia without motor block. Genicular blocks are excellent for chronic pain.
Ankle (Tibiotalar)	Tibial sensory branches, deep peroneal, superficial peroneal, sural, saphenous	Ankle block (5 nerves)	All five nerves are purely sensory at the ankle level — ideal for analgesia without motor impairment.
Foot (Tarsal, Metatarsal)	Plantar nerves, dorsal digital nerves, sural, saphenous	Ankle block + selective plantar/dorsal nerve blocks	Use selective blocks for localized forefoot or hindfoot pain. Ankle block covers the entire foot.
Toes (digital joints)	Proper digital nerves (paired plantar + dorsal)	Digital nerve blocks	Pure sensory; perfect for toe joints synovial biopsies

PENG, pericapsular nerve group block; LFCN, lateral femoral cutaneous nerve; FICB, fascia iliaca block; ACB, adductor canal block; IPACK, infiltration between the popliteal artery and capsule of the knee.

The best motor-sparing NB for analgesia at the shoulder is a combination of a suprascapular NB plus an axillary NB, with or without a low-volume superior trunk level block. This is the ideal combination for shoulder analgesia without phrenic palsy. Superior trunk adds coverage with minimal motor impact (21–26) (Table 3).

The axillary brachial plexus (BP) NB is adequate for forearm and hand anesthesia.

However, it requires musculocutaneous NB supplementation for full forearm anesthesia. It still reliably blocks a wide range of nerves-median, ulnar, radial, medial cutaneous of the arm and medial cutaneous of the forearm. It has no risk of phrenic nerve palsy or Horner’s syndrome, and the pneumothorax risk is minimal (21–26) (Table 3).

A motor-sparing NB for analgesia at the elbow would be a combination of a lateral antebrachial cutaneous NB plus a medial antebrachial cutaneous NB, plus a superficial radial NB, with or without a low-volume median or ulnar sensory-only blocks. This provides circumferential elbow analgesia while preserving biceps, triceps, and forearm flexor/extensor strength (21, 27) (Table 3).

A motor-sparing NB for analgesia at the wrist and/or hand would be a combination of a median nerve, ulnar nerve, and superficial radial nerve blocks, all at wrist level or an axillary BP NB. Since proximal muscles are spared, there is a minimal motor compromise of the thenar muscles with the median nerve block and of the interossei and hypothenar muscles with the ulnar nerve block. This combination is excellent for analgesia and covers the entire hand (28, 29) (Table 3).

The best motor-sparing NB for analgesia at the fingers or thumb joints is a paired digital nerve block (at the volar and dorsal web interdigital space), or the blocks used for wrist/hand analgesia, excluding the ulnar nerve block if aiming for the thumb joints. Digital blocks are excellent for finger joint synovial biopsies. For fingers and thumb, digital blocks are the most targeted and fully motor-sparing (28, 29) (Table 3).

A motor-sparing NB for analgesia at the hip would be a combination of a pericapsular nerve group block (PENG), plus a low-volume supra-inguinal fascia iliaca block (FICB), plus a lateral femoral cutaneous (LFC) NB, with or without an obturator articular branch block. The PENG targets the high articular branches of the femoral, obturator, and accessory obturator nerves, making this block much less prone to motor blockade than femoral nerve or complete infra-inguinal fascia iliaca blocks (30–33). A supra-inguinal FICB adds broader plexus coverage with minimal quadriceps weakness and provides good coverage of femoral and LFC nerves, with partial obturator nerve coverage. The LFC NB, if added, covers the lateral hip and thigh. The obturator articular branch block should be considered in case of medial hip pain (30–33) (Table 4).

The best motor-sparing NB for analgesia at the knee is a combination of adductor canal block (ACB) plus an iPACK (infiltration between the popliteal artery and capsule of the knee) block, with or without genicular nerve (GN) blocks—superior medial, superior lateral, and inferior medial. This combination has as primary sensory targets the saphenous nerve, the nerve to vastus medialis, the genicular branches, and the posterior capsule sensory branches. ACB targets the saphenous nerve and articular branches and preserves quadriceps strength. iPACK provides posterior capsule analgesia without motor block, and genicular blocks are excellent for chronic pain (34, 35). Adductor canal and iPACK blocks have increasingly become the preferred NB for the anterior and posterior knee, respectively, due to lesser motor involvement and because only sensory branches of the sciatic nerve are involved in the iPACK block (34, 35). Procedures in the medial leg are also preferably conducted using the ACB. Blocking the posterior division of the obturator nerve at the proximal medial thigh also provides analgesia to the popliteal region, without motor compromise. In case genicular nerve blocks are considered, there is no consensus on the ideal number or combination of GN to be targeted, but most practitioners avoid targeting the inferior lateral GN to not damage the common peroneal nerve, which could result in a disabling iatrogenic foot drop, and recommend targeting the remaining GN (36) (Table 4).

A motor-sparing NB for analgesia at the ankle should include all five primary sensory targets, namely the tibial sensory branches, the deep peroneal, the superficial peroneal, the sural, and the saphenous nerves, the so-called ankle block. All five nerves are purely sensory at the ankle level, being ideal for analgesia without motor impairment (27, 35) (Table 4).

A motor-sparing NB for analgesia at the foot (tarsus, metatarsus) would be an ankle block or a selective plantar/dorsal nerve block. The primary sensory targets will be the plantar nerves, the dorsal digital nerves, the sural, and/or the saphenous nerve. The ankle block covers the entire foot, but selective blocks should be preferred for localized forefoot or hindfoot pain (27, 35) (Table 4).

The best motor-sparing NB for analgesia at the toe joints will be the digital nerve blocks, targeting the proper digital nerves (paired plantar + dorsal). This option is a pure sensory one, being perfect for toe joints synovial biopsies (27, 35) (Table 4).

Specific adverse events (AE) of LA NBs include peripheral nerve injury, muscle weakness or paresis, Horner syndrome, epidural or spinal injection, vessel injury, pneumothorax, and systemic toxicity. Peripheral nerve injury, resulting in neuropathic symptoms, is the most common AE of NBs and can be caused by mechanical, neurotoxic, or ischemic trauma to the nerve. The frequency of neuropathic symptoms shows a considerable variability in the long-term, from < 1 to 24%, according to the published series (3). On the other hand, systemic toxicity may occur when LA is inadvertently given IV, resulting in a life-threatening event with hypotension, arrhythmia, cardiac arrest, seizures, and loss of consciousness (13, 14, 16). Besides, a reduction in LA single or daily dose is assumed if any of the following are present: underweight, pregnancy, old age, and liver, kidney, or heart insufficiency (37).

However, doubts still remain regarding the ideal LA volume and concentration, nerves to target for each region, timings, and patient selection criteria (37).

#### Chemoneurolysis

Chemoneurolysis is a dose-dependent destruction of a nerve, resulting in necrosis, Wallerian degeneration, and a complete conduction block in all fibers contained within that nerve. A 35–60% alcohol, 3–12% phenol, and 50% glycerol are the most common agents used for this purpose and are used in the approach of intractable neuropathic pain and spasticity. However, because all these substances are non-selective agents, having the potential for irreversible off-target tissue damage, namely in non-nerve tissues, they are not approved by the FDA for the treatment of neuropathic pain and should be a therapy of last resort and not to be used in the routine care of non-cancer patients with chronic pain (38).

High-concentration capsaicin 8% topical system, although a chemoneurolytic agent, is not toxic for surrounding tissues due to its selectivity for transient receptor potential vanilloid 1, which is only expressed in nerve fibers. However, being a transdermal therapy will not be further covered in this review (38).

A chemoneurolytic block is usually administered with guidance from ultrasound, fluoroscopy, electrical nerve stimulation, or a combination of these techniques. Usually, before performing a neurolytic block, a confirmatory LA NB is conducted to assess the correct location of the intended procedure, and after neurolysis, another local injection of LA plus a CS is usually administered.

Alcohol damages nerves by nonselectively denaturing proteins, by extracting cholesterol, phospholipids, and cerebroside from the neural membranes, and by precipitating mucoproteins and lipoproteins, leading to a retrograde Wallerian degeneration of nerve fibers. Phenol causes nonselective nerve destruction by denaturing proteins and by causing the loss of cellular fatty content, separating the myelin sheath from the axon and causing axonal edema and Schwann cell dissolution (38).

Complications of chemoneurolysis include trauma to the injection site tissue, vascular injury, muscle fibrosis or necrosis, dysesthesias, neuritis, “anestesia dolorosa” through deafferentation pain after nerve lesion, permanent weakness and, resulting from systemic absorption, also central nervous system effects and cardiovascular, pulmonary, liver, or renal compromise (38).

#### Percutaneous electrical nerve stimulation

Percutaneous electrical nerve stimulation (PENS) consists of the application of an electric current to the vicinity of peripheral nerves in musculoskeletal structures, aiming to decrease pain signals or motor stimulation. The electric current is administered to the target nerve through a percutaneous lead, previously US-guided, placed and left close to the nerve, with the lead connected to an external pulse generator, attached to the skin. US-guidance eases the procedure of lead implantation by providing a direct view of the nerve while preventing neurovascular damage. In detail, under real-time US visualization, an introducer needle preloaded with a stimulating probe is inserted into the target. Once optimal stimulation parameters and lead position are obtained, the stimulating probe is replaced by the open-coil peripheral nerve stimulation (PNS) lead via the introducer needle. Lastly, the needle is removed as the PNS lead is deployed under continuous US visualization (39–41). The electric current is most commonly biphasic with frequencies ranging from 2–5 Hz to 80–100 Hz, having a pulse width ranging from 250 to 500 ms. The inner lead remains implanted for up to 60 days, until it is removed with gentle traction. However, permanent devices may also be used (39).

PENS is used to reduce nociceptive and neuropathic pain but also to improve sports performance (39, 40). It is postulated that the mechanism underlying the PENS effect is explained by the gate control theory, which suggests inhibition of nociceptive inputs from small-diameter nerve fibers through the electric activation of the large-diameter sensory nerve fibers. However, other mechanisms of PENS action may include inhibition of local nociceptive neurotransmitters, alteration in ion channels, and endogenous opioid activation pathway (39).

PENS is indicated in the approach of peri-operative and neuropathic pain and also in other cases of recalcitrant chronic pain, including post-amputation pain, hemiparetic shoulder pain, radiculopathies, and complex regional pain syndrome (40–42).

Pain is the most common adverse event of PENS, occurring in up to 25% of cases. Pain is much more frequent with internal pulse generators than with external ones, and that explains why the former have been deprecated in favor of the latter.

Complications of PENS may be hardware-related or biological. In the first group, we include lead migration, breakage, and disconnection. In the second group, we include infection, seroma, hematoma, and nerve damage (41, 42).

Multiple technical issues remain to be answered regarding optimal lead location, insertion technique, stimulating protocol, and prevention of lead dislodgement and breakage. When dealing with PENS, consideration must be given to the ergonomics of pulse generator placement and patient comfort. It seems critical that lead wires do not cross large joints because of the risk of lead migration and that at least 3–4 cm of the lead should be inside the body before exiting at the skin, to prevent inadvertent migration, removal, or fracture (43, 44). Key take-home messages related to NBs:

- LA NB – LA block Na-specific ion channels on neuronal cell membranes, inhibiting signal conduction; motor-sparing NB can be used to approach upper limb and lower limb pathologies
- Chemoneurolysis – substances like ethanol, phenol, or glycerol promote a dose-dependent destruction of a nerve, resulting in necrosis, Wallerian degeneration, and a conduction block
- PENS – the application of an electric current to the vicinity of a peripheral nerve, aiming to decrease pain signals or motor stimulation

### Ozone

Therapeutic ozone (O_3_) is a mixture of oxygen (O_2_) plus ozone (O_2_O_3_), composed of at least 95 % O_2_ and no more than 5 % O_3,_ and is usually administered as a gas, in doses ranging between 15 and 40 micrograms/mL. Using US, it appears as a hyperechoic area when it enters the target zone (45).

O_2_O_3_ has anti-inflammatory, antioxidant, analgesic, and immunomodulatory effects, through a balanced production of reactive oxygen species, like hydrogen peroxide, which can damage cell components, counteracted by antioxidant response elements, whose production is induced by small repeated oxidative stresses. O_2_O_3_ stimulates the synthesis of chondrocytes and fibroblasts, promotes activation of cellular metabolism, reduces proinflammatory prostaglandins and algogenic compounds, increases immunosuppressive cytokines, serotonin, and endogenous opioids, and improves tissue O_2_ supply through hemorheological action, vasodilatation, and angiogenesis stimulation. It also exhibits virucidal and bactericidal actions, with applications in wound healing, ischemic disorders, infections, and chronic painful and inflammatory conditions (45–47).

Common indications in musculoskeletal disorders include axial pain (with or without radiculopathy), shoulder impingement, enthesopathies (epicondylosis, plantar fasciitis), myofascial pain syndrome, fibromyalgia, and OA, and is usually administered in doses between 20 and 40 μg/mL, by intramuscular, intra-discal, intra-foraminal or intra-articular route, or through injections in soft tissues, with many of these preferably USG (45, 47, 48).

Some RCTs, SLR, and MA have shown favorable results in pain management in low back pain with herniated disk and knee osteoarthritis, versus placebo or active comparators (45–54). In one of these studies, the combination of ozone with HA produced the best outcome in knee osteoarthritis (KOA), compared to each of the products isolated (54). In two other studies, ozone was comparable to hyaluronic acid in one, whereas in another the beneficial effect of ozone lasted longer than triamcinolone acetonide (55, 56). Compared to placebo, ozone was better in three systematic reviews and meta-analyses in KOA, but inferior to HA in the other two (57–61). However, an umbrella review of eight systematic reviews of randomized clinical trials on the efficacy of ozone in improving pain and function in KOA displayed a substantial risk of bias (62). The majority of the systematic reviews concluded that ozone enhanced short-term (3–6 months) pain management in mild to moderate KOA, in which ozone appeared superior to placebo, but not surpassing intra-articular injections of other substances, namely HA (62).

A systematic literature review on ozone therapy for neck pain found two randomized controlled trials (RCTs) and 5 non-randomized observational studies. RCTs focused on the treatment of neck myofascial pain syndrome. Two other studies investigated intradiscal ozone nucleolysis in patients with cervical herniated discs. The remaining three studies dealt with paravertebral ozone intramuscular injections in undifferentiated neck pain. The RCTs showed that ozone was not better than LA injections. The remaining five studies all showed positive results, but they were all uncontrolled (48).

Ozone was compared to steroids as local injections in lateral epicondylosis. No differences were found between groups (63). In fibromyalgia, one uncontrolled study showed benefit in pain and functional status (64). A double-blind randomized controlled study compared local injections of a steroid with ozone in plantar fasciitis. Both groups improved, but steroids showed better results in the short term, although no difference between groups after 3 months (65).

Contraindications for ozone therapy include glucose 6-phosphate dehydrogenase deficiency, pregnancy, uncontrolled hyperthyroidism, and severe cardiovascular disease (39, 41).

AE with ozone therapy are usually mild and transient and may include local discomfort, dysphagia, and sore throat. Nevertheless, rare but serious AE may occur, including sepsis, gas embolization, stroke, and death (45–48).

Notably, evidence about O_2_O_3_ efficacy in musculoskeletal pain management is still lacking due to recruitment bias, low-quality studies, and heterogeneity in treatment protocols (48, 62).

Key take-home messages related to ozone therapy:

- O_2_O_3_ – produces reactive oxygen species, which trigger small repeated oxidative stresses with consequent production of antioxidant response elements
- O_2_O_3_ – increases local oxygen supply and has anti-inflammatory, antioxidant, analgesic, and immunomodulatory effects

### Platelet-rich plasma and derivatives

Autologous Peripheral Blood-Derived Orthobiologics (APBDO) refers to a subgroup of orthobiologics derived from the patient’s own blood and includes: (1) PRP and derivatives—platelet-rich fibrin (PRF), jellified PRP or plasma gel, platelet-rich releasate/supernatant, platelet-rich lysate, allogenic PRP, and PRP combined with other molecules or solutions; (2) autologous conditioned serum (ACS), derived from incubating venous blood within specialized glass spheres, which produces high levels of anti-inflammatory cytokines through monocyte and platelet activation; (3) gold induced cytokine (Goldic), which involves incubating whole blood with gold particles; (4) plasma rich in growth factors (PRGF), which is formulated by activating erythrocyte poor and leucocyte poor-PRP (LP-PRP) with CaCl_2_; (5) growth-factor concentrate (GFC), which is prepared by incubating whole blood with an external platelet activator; (6) autologous protein solution (APS), formulated by exposing leucocyte rich-PRP (LR-PRP) to polyacrylamide beads; (7) hyperacute serum (HS), which is prepared by squeezing serum from a PRF clot, using pressure or centrifugation; and (8) autologous conditioned plasma (ACP), which is a single-spin LP-PRP formulation. Compared to the other APBDO, PRP has the highest levels of GFs and cytokines, but also the highest levels of the pro-inflammatory ones (66–73).

PRP is currently defined in terms of a few critical variables and preparation protocols that should be mentioned, and include: total volume injected; concentration/number of platelets in the PRP vs. whole blood, and capture efficiency of the platelets from blood; WBCs number, from minimal to below or above the baseline; presence or absence of red blood cells; GF concentration; anticoagulant used and method of activation, if any; single vs. double spinning centrifugation, centrifugation time and G-force; fresh vs. frozen PRP.

Usually, to prepare PRP, also known as a first-generation blood product, a 40–60 mL volume of blood is collected from the patient. PRP is then delivered in a 3–6 mL volume, obtained from anticoagulated whole blood after a single or double spinning centrifugation, and seemingly the ideal platelet count should be above three to five times the whole blood platelet count, according to authors (74–78). Additionally, PRP can be obtained through standard lab protocols, through kits that allow clinicians to specify total volume and leucocyte concentration or, less often and still in preliminary studies, through PRP lysates from bovine origin or allogenic PRP derived from umbilical cord blood (74–78). In more advanced centers, PRP may also be obtained through apheresis, which provides a leucocyte-depleted PRP with the highest platelet yield of improved quality, compared to venous blood-derived PRP (77). Patient variability that may influence PRP quality includes age, gender, comorbidities, and medication, with higher levels of GFs found in younger subjects and in females. The desired outcomes of PRP preparation are high yields of platelets, enhanced levels of platelet-derived GFs in activated PRP, product sterility, and reproducibility (77).

In the case of PRP obtained from autologous whole blood, it is mandatory to ensure that the patient has no thrombocytopenia and that platelet viability and fragility are considered through the whole processing system, keeping in mind that even a hard container used during centrifugation can prematurely activate platelets, in opposition to a soft one. Besides, PRP should be kept in small-diameter tubes with caps to minimize contact with the atmosphere, as the information about the optimal anticoagulant to use is still contradictory in the literature. For some authors, citrate-dextrose-A is the best anticoagulant to consider, while for others is ethylenediaminetetraacetic acid (EDTA), allegedly because it allows higher platelet counts and volumes and prevents more efficiently a possible spontaneous platelet activation (69, 74, 75).

After blood sampling and coagulation, the third step in PRP preparation is centrifugation. The first spin centrifugation aims to obtain three layers from the collected blood, which are from top to bottom, the plasma layer, composed of platelet-rich and platelet-poor plasma, the intermediate layer or buffy coat, composed of white blood cells (WBC), and the bottom layer, composed of red blood cells (RBC). The double spin centrifugation aims to separate platelet-rich from platelet-poor plasma (74, 75, 78–81).

PRP can be delivered in 2 forms: LR-PRP or buffy coat-based method, and LP-PRP or plasma-based method, with neutrophil numbers above the blood baseline, or minimal, respectively. For obtaining leucocyte-rich PRP (LR-PRP), the upper and middle layers are collected, the first spin is stronger and longer and a trace of RBC is acceptable.

For obtaining leucocyte-poor PRP (LP-PRP), only the upper 2/3 to 3/4 of the plasma layer are aspirated, staying away from the buffy-coat, the first spin is softer and shorter and visible RBC are avoided. LR-PRP induces predominantly catabolic and proinflammatory changes and LP-PRP predominantly anabolic ones, possibly through M1 and M2 macrophages, respectively (79, 80). Seemingly, LR-PRP is better for tendinopathies, and LP-PRP is better for intra-articular injections (OA), although different PRP preparations have not been directly compared, but in lateral epicondylosis, in which positive results were equivalent for both preparations (74, 75, 80–82).

RBC in PRP can release macrophage migration inhibitory factor, a cytokine that blocks cell migration, suppresses fibroblast growth, promotes inflammation, and disrupts local cell function (eryptosis). This is why adequate centrifugation and preparation processes should reduce or even eliminate the presence of RBCs in PRP preparations (78).

Differences in G-force and centrifugation time result in significant differences in yields, purity, concentration, viability and activation status of the isolated platelets (78). Although the double-spinning centrifugation can generate a higher platelet count, longer times of spinning may prematurely activate platelets and they may also be lost in the process due to lysing (75, 79). There is also debate about the ideal centrifugal force, with the first spin usually using lower forces than the second one (74, 75). A study that compared three different spinning rates (1,000–3,600 rotations per minute) found that lower spinning rates had higher platelet yields, hypothesizing that higher rates could cause platelet clumping and disintegration (83). The use of fixed-angle versus horizontal rotators also needs further research in terms of the final product quality (77).

PRP may be fresh or frozen. In the first case, it may be kept under normal or controlled temperature but should be injected as soon as possible after processing; otherwise, activity may be lost. Notably, when using fresh PRP, preactivation is not recommended before injection, only after freezing and thawing (74). On the other hand, if a treatment requires several doses, PRP can be preserved under freezing conditions and then activated with thrombin or calcium chloride, through photoactivation or after exposure to electric pulses (78, 79). However, the frozen/thawing method may negatively affect the final quality of the product (74, 77).

There are several classification systems for PRP preparation, including the PAW, PLRA, DEPA and MARSPILL classifications, but there is a lack of a single, agreed-upon one, which would make comparison across studies possible or at least easier (84–90).

PRP can be delivered isolated (from one single injection up to three injections, one every 3 weeks), or in combination with platelet-rich fibrin, plasma gel, HA, MSC, CS, LA, and DN, being the association with DN and LA the most common one (83, 91–95). Volumes of 3–6 mL for soft tissues and of 6–10 mL for joints are usually recommended per injection (81, 93). It can be delivered into soft tissues (entheses, ligaments, muscles, scars), or through the intra-articular or intra-spinal (intradiscal, epidural, intrathecal) routes.

PRP acts through the release of growth factors (GFs), adhesive proteins, chemokines, clotting factors and inhibitors, integral membrane proteins and immune mediators essential for cells to proliferate and differentiate in the healing process (73). These factors act by promoting chondrocyte, mesenchymal stem cells, tenocytes, and osteoblasts proliferation, also having an anti-inflammatory effect. Besides, PRP healing properties derive from the enhancement of adhesion, recruitment, proliferation, migration, and differentiation of stromal cells. It increases HA concentration, restores angiogenesis function, decreases synovial membrane hyperplasia, and participates in tissue remodeling, matrix production, chondrogenic differentiation, and production of proteoglycan and type II collagen, improving the metabolic function of damaged structures (79, 96). The main GFs released from platelets’ α-granules include: platelet-derived GF, transforming GF β, epidermal GF, connective tissue GF, vascular endothelial GF, insulin-like GF, platelet factor 4, brain-derived neurotrophic factor, angiopoietin-1, keratinocyte GF, hepatocyte GF, and stromal cell-derived GF. Besides, platelet-derived 5-HT exerts immunomodulatory and anti-nociceptive actions (78, 97).

NSAIDs and CS are to be avoided when PRP is used, because they affect the platelet secretome release, inhibiting the PRP therapeutic effect, and PRP use should be hindered in the vicinity of malignant or dysplastic tissues (78, 79, 81, 92). When using antiplatelet drugs, it is advisable to consider a 1-week washout period (78).

Common indications for PRP include bone graft and non-union fractures, cutaneous wound healing, tendinopathies, enthesopathies, muscle, meniscal, and ligament injuries, adhesive capsulitis, and OA (75, 81, 82, 97).

Most RCTs, SLR, or MA evaluating the role of PRP in the treatment of rotator cuff tendinopathy show better results over placebo, physiotherapy, or CS, in the mid and long-term, while some studies show CS to be superior in the short-term. Conflicting results occur in subacromial impingement comparing PRP to CS, and worse outcomes were found in calcific tendinosis compared to barbotage, but only in the short-term, not in the mid-term (67, 81, 95, 98). A SLR on intra-articular injections of PRP in frozen shoulder, analyzing PRP alone versus hydrodissection or CS, showed better clinical and functional outcomes with PRP in all studies, except in one in which results were comparable to CS. Other studies in frozen shoulder revealed that PRP was superior to placebo and as effective as physiotherapy (76, 99). Two RCTs in gleno-humeral OA showed that PRP was superior to CS and equal to HA, in the long-term in gleno-humeral OA (97).

Several SLR and MA including thousands of patients in RCT, comparing PRP to saline placebo, HA, ozone and/or CS in KOA, concluded that PRP had the best overall outcome in pain relief, physical function and in other patient reported outcomes (PRP) in diverse timepoints (3, 6, and 12 months) and in all stages of KOA, producing the longer lasting effects with a low risk of adverse events (97, 100–102). However, a combination of PRP plus HA showed better results than any of the injectables isolated (100). Besides knee OA, there seems to be moderate to high-quality evidence to support the use of LP-PRP for knee chondropathy and moderate evidence of LR-PRP for patellar tendinosis, particularly in the long-term, with a lack of efficacy, conflicting results, or insufficient data to support its use in meniscal lesions, muscle injuries, acute or non-union fractures, as well as in ligament lesions (81, 97).

In 4 RCTs in hip OA, PRP improved pain and PRO up to 1 year, but it was not superior to HA. Also, PRP didn’t perform differently from other injectables or placebo. In trochanteric pain syndrome, PRP produced mainly positive outcomes, but conflicting results can also be found (97, 103, 104).

A few studies concerning PRP in tibiotalar OA produced conflicting results, but PRP seemed to deliver positive results as an added procedure to surgery in osteochondral defects (81, 97, 105, 106).

In plantar fasciitis, PRP produces positive results, similar or superior to CS or extra-corporeal shock wave therapy, with the benefits of PRP lasting longer (81, 97, 105, 106). Likewise, when comparing PRP with phonophoresis plus kinesiotaping, the former produced better results in the mid-term, regarding pain, function, and fascia thickness, but not in the short-term, and PRP seemed to be ineffective in Achilles tendinopathy (75, 97, 105, 107).

The majority of studies favor PRP over CS in lateral epicondylosis, regarding pain and function in the intermediate term. However, CS may be superior in the short term. Some case series have shown good results of PRP in ulnar collateral ligament injuries, as well (67, 75, 81, 82, 108).

At the hand, a SLR and MA revealed better results in carpal tunnel syndrome with PRP compared to CS in the mid-term follow-up. In De Quervain’s disease, CS were better in the short-term and PRP better in the mid-term, with conflicting results of PRP in carpal-metacarpal joint osteoarthritis (97).

Only a few preliminary promising results exist about the utility of PRP in sacro-iliac joints and in intradiscal, transforaminal, and facet joints in the spine (97).

In summary, level I research supports the use of PRP in knee and hip OA. Limited data exist on PRP application on spine, hand, and shoulder OA, and no studies support its use in elbow or ankle OA. Level I research supports PRP use in lateral epicondylosis, trochanteric pain syndrome, plantar fasciitis, carpal tunnel syndrome, and rotator cuff tendinopathy (97).

Local fibrosis/scar and thrombi formation are PRP-specific AE (66, 77, 91).

Despite positive results, heterogeneity in preparation, concentration and delivery protocols are still barriers to clinical applicability and research regarding PRP, including traditional versus commercial kits, temperature, centrifugation protocols, time from plasma isolation, effect of leucocyte, red blood cells and platelet concentration and activation, fibrin presence or absence, activation methods, storage aspects, dosage/volume, number and timing of injections and absence of an informative unified classification. Besides these unmet needs, discontinuation of antiplatelet agents and NSAIDs before treatment is still debated, as well as the possible deleterious effect of adding LA or CS to the injectate, or the eventual added benefit of combined therapy (73–75, 92, 95).

To prepare PRF, also known as a second-generation blood product, peripheral blood is harvested and centrifuged quickly enough to avoid coagulation, given that no anticoagulant is added previously. PRF, which contains the same diversity of GFs as platelets, is a fibrin-matrix polymer that incorporates platelets, leucocytes, cytokines, and even circulating stem cells. It provides a scaffold to improve cell migration and growth, having angiogenic, osteogenic, and anti-bacterial capacities, enabling the release of molecules over an extended period of time, while promoting tissue regeneration and healing (69–72, 93). Several formulations of PRF exist: leucocyte, advanced, titanium, and injectable PRF. Injectable PRF is typically prepared in vacuum tubes, with no additives and using slower and shorter centrifugation protocols (70). In opposition to injectable PRF, the remaining modalities of PRF can only be used in scaffolds, because of their compact 3D structure (69–72). Due to its adhesive properties, PRF allegedly stays longer in targets (osteoarthritic joints or tendinopathies), sustaining a prolonged GF and cytokine delivery (69, 72). Combining PRF with PRP and HA is also being proposed (“power mix gel”) (94).

Plasma gel is obtained by heating the plasma to 70°C for 15–20 min. This process polymerises plasmatic proteins, forming a solid aggregate cross-linked with fibrin networks, which have been reported to be a rich source of GFs and to present chemotactic, migratory, and proliferative activity. Plasma gel is proposed to be combined with LR-PRP and HA, allegedly enhancing the regenerative properties and clinical outcomes of PRP (72).

Platelet-rich releasate/supernatant (PRR) is prepared by clotting PRP ex vivo. The released serum is incubated at 37°C and centrifuged (66).

Platelet-rich lysate (PRL) is prepared through several freezing-thawing cycles or sonication of the liquid PRP, being a cell-free platelet derivative. In the case of PRL, the intracellular components of the platelets have been released and collected as an injectate. Contrary to PRR, PRL contains high molecular weight plasma proteins, which can produce different angiogenic and inflammatory outcomes. Both PRR and PRL can be preferred over pure PRP to contour the PRP thrombogenic nature. However, in the case of intra-articular injections, PRP may be preferred because it allows a better confinement of the released protein pool (66).

ACS is an autologous serum enriched in products released from monocytes and platelets. It is produced by incubating venous blood in a specialized ACS-processing syringe whose inner walls, formed by CrSO4-coated glass beads/spheres, trigger blood cells to produce anti-inflammatory cytokines after centrifugation, mainly IL-1Ra, but also IL-4, IL-10, chemokines, and GFs (96). ACS aims primarily to modulate the inflammatory environment within the joint, which seems to be related to exosome expansion during the incubation process, while PRP focuses more on stimulating tissue repair and regeneration (73, 91). Contrary to PRP, it does not contain any additives (e.g., anticoagulants) and has a different cytokine profile. We must keep in mind that any systemic inflammation at the time of blood sampling may shift the cytokine profile in favor of pro-inflammatory factors (67). There are already a few RCTs that favor ACS over placebo or HA in knee OA and over CS in lumbar radiculopathy or neck pain (67). SLR about ACS in KOA showed similar or better results with ACS compared to PRP, with minimal AE reported (68, 109, 110). ACS also showed positive results in hip OA in two retrospective non-blinded, non-randomized studies, in gleno-humeral OA, and in epicondylosis in a prospective, non-comparative pilot study (111–113). However, the lack of standardization in preparation, dosage, and administration schedules precludes clear recommendations on the use of ACS (110).

Besides ACS, for the remaining APBDO, there are mostly only scarce and preliminary positive results in pain and function. In shoulder disorders, PRL, ACS, and PRGF showed positive results in rotator cuff tendinopathies, PRGF in delayed union fractures of the clavicle, and APS in patients with subacromial impingement syndrome (112). PRL also showed positive results in epicondylosis in two low-quality, non-comparative studies (110, 112). Mostly in KOA, HS, ACP, APS, GFC, and Goldic have been showing positive results in pain and function, and no adverse events in preliminary studies (68, 113).

Key take-home messages related to APBDO:

- APBDO - a subgroup of orthobiologics (a branch of regenerative medicine applied to the MS system) derived from the patient’s own blood
- APBDO - includes PRP and derivatives, and other molecules
- APBDO - act through the release of GF, adhesive proteins, chemokines, clotting factors and inhibitors, integral membrane proteins, and immune mediators
- PRP - acts by inducing catabolic and proinflammatory changes (LR-PRP) or anabolic ones (LP-PRP), aiming at regeneration and natural healing

### Prolotherapy

Prolotherapy (PRL) is usually performed with hypertonic dextrose, but other agents, like sodium morrhuate or phenol-glycerine-glucose, may also be used, usually in conjunction with a LA, in a 1:1 to 1:4 ratio before injection (73, 91).

Common indications include OA, tendinopathies, enthesopathies, ligament lesions, axial pain, and MPS (107, 108).

It is thought that PRL acts through pain modulation, prochondrogenic effects, and by initiating a local inflammatory cascade with GFs release (within hours to days), leading to tissue proliferation (within days to weeks) and remodeling (within weeks to months) (91, 114, 115).

Most authors favor a 3-injection regime, 0.5–10 mL, of 15% DW for tendons and 25% for joints, but it remains to be known what the optimal dextrose concentration or the best PRL agent or injection number, volume, and interval (106, 108). However, dextrose PRL was found to be safe, with only mild and transient adverse events, like local pain, swelling, and bruising or, rarely, neuropraxia (112, 114–116).

A SLR of dextrose PRL for chronic MS pain included 14 RCTs (all very high-quality studies), 18 case-series studies, and 1 case-control study, referring to tendinopathies (17 studies), arthropathies (8 studies), axial or pelvic pain (7 studies), and MPS (1 study). PRL was found to be superior to controls in Osgood-Schlatter disease, TMJ dysfunction, KOA, and finger OA (all LoE1). Also, positive results in coccygodynia, lateral epicondylosis, rotator cuff, adductor, Achilles, and patellar tendinopathies, plantar fasciitis, and low back pain, although with a LoE4, have been reported. The authors concluded that dextrose PRL was supported in the treatment of tendinopathies, knee and finger OA, and in axial pain, in patients who didn’t respond to other conservative therapies (114).

A SLR of PRL on chronic low back pain (LBP) of at least 6 months duration with failure of other conservative approaches, including 6 RCTs, 3 prospective, and 3 retrospective studies, was performed (117). The authors concluded that PRL was an effective therapeutic option for this subgroup of patients. However, concerning the use of other substances like sodium morrhuate, the poor quality of the studies, the selection of patients based on symptoms and not on specific diagnosis, and the uncontrolled dry needling effect were all factors decreasing the LoE of this therapeutic modality (117). The main AE reported related to PRL were transient pain, swelling, or stiffness at the injection site and, less often, headache, nausea, diarrhea, or minor allergic reactions (117, 118).

A review of the literature addressing chronic LBP treatment in the context of believed lumbar instability, with spinal ligaments (posterior ligamentous complex plus the facet joints capsular ligaments), as underlying culprits, showed better results with PRL (phenol-glycerine-glucose) compared to LA (118). Also, injection of sacro-iliac ligaments using PRL was better than intra-articular sacro-iliac injection with CS, in sacro-iliac pain (118).

Concerning PRL for upper limb disorders, RCTs showed that PRL was superior to LA or CS in the treatment of trapeze-metacarpal joint OA, superior to NS or CS in lateral epicondylosis, and superior to usual care in non-traumatic rotator cuff tears. However, all these studies had small numbers, used non-standardized protocols, and not all used patient-reported outcomes (115).

A SLR of RCTs comparing dextrose PRL to any kind of intervention, including placebo, in KOA, concluded that, although with a high risk of bias, PRL was superior to placebo, equal to ozone and to physical therapy, equal or superior to botulinum toxin, equal or inferior to PRP at 6 months, and worse than HA or pulsed RF. It was concluded that PRL could be considered in knee OA with limited alternative therapeutic options, but more high-quality RCTs were warranted (116).

Another SLR concerning 8 RCTs of dextrose-PRL in KOA showed that PRL-treated patients improved in comparison to baseline and that it was better than NS, but when compared to other substances, the results were not uniform—PRL equal to botulinum toxin and worse than PRP or autologous conditioned serum (119). Contradictory results were also found with regard to HA and erythropoietin. However, most studies had small patient numbers (120).

A third SLR and MA regarding D-PRL in KOA, compared it to NS, sterile water, or exercise programs. Dextrose PRL surpassed comparators in terms of pain and knee function. Five studies and 319 patients were included, with a mean follow-up of 22, 8 weeks, with only mild and transient side-effects (119).

A SLR and MA on the effects of dextrose-PRL in lateral epicondylosis, compared dextrose-PRL to DN, NS, LA, CS, PRP, AWB, or HA injections, and to several modalities of physiotherapy. Overall results revealed superiority of dextrose-PRL compared to other active controls at 12 weeks, regarding pain improvement and function (119). Three hundred and fifty-four patients were included in eight studies, with an overall quality ranging from low to moderate (121).

A SLR aiming to evaluate dextrose PRL in the treatment of plantar fasciitis included 6 studies in a total of 388 patients. PRL showed to be superior to placebo or exercise, inferior to CS or extra-corporeal shock wave therapy (ECSWT) in the short-term, but superior to CS in the long-term (122). Another SLR, including 8 studies and 449 patients that evaluated dextrose PRL in the short-term in chronic plantar fasciitis, showed it was better than exercise and placebo, but not superior to CS or ECSWT (123).

Sclerotherapy versus dextrose PRL was evaluated in a SLR that included 10 studies. Both methods showed positive results in reducing pain and in improving function in chronic patellar tendinopathy, although it was recognized that more RCTs with larger numbers and longer follow-up were needed (124).

Key take-home messages related to prolotherapy:

- PRL – is usually performed with hypertonic dextrose, but other agents like phenol-glycerine or sodium morrhuate may also be used, in conjunction with LA
- PRL – acts through pain modulation, prochondrogenic effects, and by initiating a local inflammatory cascade with GF release, leading to tissue proliferation and remodeling

### Radiopharmaceuticals

Radiopharmaceuticals (RPH) commercially available and approved in Europe include [90 Y] yttrium citrate (for the knee), [169 Er] erbium citrate (for finger and toe joints), and [186 Re] rhenium sulfide (for all other joints). [165 Dy] Dysprosium hydroxide is also used in the USA for large joints.

Radiosynovectomy (RS) must be conducted in a dedicated facility for appropriate handling of radioactive substances and waste, and β-emitting isotopes are only for intra-articular use.

LA and CS are usually coadministred to bridge the lag to the RS effect, to avoid the RPH linkage, and to decrease the chance of radiation synovitis. However, small joints may not allow coadministration of LA or CS.

Radionuclide particles are gobbled by the phagocytizing subsynovial cells, resulting in a decrease in the inflammatory response, inhibition of cell proliferation, fibrosis, and cell death (125, 126).

Common indications for RS include synovitis (RA, PsA), osteochondromatosis, hemophilic hemarthrosis, thumb base osteoarthritis, and pigmented villonodular synovitis, sometimes as adjuvant treatment to surgical synovectomy, to decrease recurrence rates, when other approaches have failed (125–129). For most of the indications above, RS shows a positive effect, although with a decrease in treatment response over time (125, 126). An interval of at least 6 months is recommended between injections in the same joint, except in hemophilia arthropathy, in which shorter intervals, up to 3 months, can be used (126).

The best candidate for radiosynovectomy would be someone with persistent monoarthritis, resistant to previous intra-articular steroid injections, and in whom septic arthritis was excluded, with an optimized systemic treatment, including bDMARDs (128). On the other hand, a poor candidate for RS would be someone with pre-existing joint damage or advanced-stage OA and with a long-standing underlying inflammatory disease (128).

Specific contraindications for RPH use include pregnant and breastfeeding women, administration to children under the age of 13, and ineffective contraception (126).

Specific AE of RS include deep vein thrombosis, radiation synovitis, skin depigmentation or necrosis, nerve or muscle damage, and avascular necrosis, with no risk added for malignancies or infection (125, 126, 128). A previous malignancy is not considered a contraindication for a RS, and when performed properly, RS has a low rate of AE and complications (128).

Key take-home messages related to radiopharmaceuticals:

- RPH - use radionuclides like yttrium citrate, rhenium sulfide, erbium citrate, or dysprosium hydroxide to treat synovitis
- RPH - act by decreasing the inflammatory response through inhibition of cell proliferation, fibrosis, and cell death

### Sclerotherapy

Sclerotherapy (ST) uses polidocanol 5 or 10 mg/mL, a vascular sclerosing agent with local anesthetic activity. Phenol may also be used in a 5% solution (91).

Common indications include tendinopathies and enthesopathies.

US identifies tendinopathies or enthesopathies with Doppler signal and repeated injections (0.1–0.2 mL each), targeting the deep-seated neovessels are performed. The rationale behind the procedure is that neovascularity is associated with the underlying mechanism of overuse tendinopathy or enthesopathy (130).

A typical treatment uses repeated cycles (up to 5) of 2 mL injection per tendon, with a maximum of 4 mL per patient per day, 3–6 weeks apart (130, 131).

Two RCTs comparing polidocanol with lidocaine plus adrenaline, in Achilles tendinosis, showed no differences between groups in one study and better results in the polidocanol group in another (131, 132). Another study in Achilles tendinosis showed no differences in the polidocanol group compared to the lidocaine one, or no differences between 5 and 10 mg/mL of polidocanol (133, 134).

A RCT compared sclerotherapy with other injectables in lateral epicondylosis. Local injection of CS produced better results at week 4, but polidocanol produced better results at week 26 (135).

A study that aimed to compare 5–10 mg/mL of polidocanol in Achilles tendinosis didn’t find any difference between groups (135).

Specific AE include tendon rupture, soft tissue edema or necrosis, and vein thrombosis (125–135). Key take-home messages related to sclerotherapy:

- ST - uses polidocanol, which is a vascular sclerosing agent with LA activity
- ST - is an USG procedure that targets tendinopathies or enthesopathies with a color Doppler signal, assuming that neovascularization is the underlying pathologic mechanism

### Thermal ablation

Nerve blocks (NB) can be performed through thermal ablation (TA), using unipolar (standard), bipolar, pulsed, or cooled radiofrequency and cryoneurolysis (136). A standard radiofrequency (RF) circuit includes a RF generator, a needle that is introduced into the patient, and a ground pad to dispel the energy (137). TA therapeutic action lies in the ability to modulate neural pathways responsible for transmitting pain signals.

Cooled RF uses a water-cooling system to lower the temperature and in cryoneurolysis a cryoprobe delivers a highly pressurized gas through a hollow tube, consisting in nitrous oxide or carbon dioxide, creating an ice ball that, although it doesn’t enter or remain in the patient’s tissues, promotes Wallerian degeneration, with no epineurium, perineurium or endoneurium effects and with the possibility of portable devices use (138). In opposition to other methods of TA, which use high temperatures (up to 80°C), cryoneurolysis applies extremely low temperatures (between minus 60 and minus 100°C) (38, 137–139).

Bipolar, pulsed RF, and cryoneurolysis have no risk of skin burns or implant device stimulation, and pulsed RF only produces pain modulation, not thermal lesions, and in fact is not a real TA method. In pulsed RF, the time that elapses between two contiguous bursts allows temperature to remain in a moderate range, and it is the electromagnetic field that it generates, rather than heat, that can result in neuromodulation—a decrease in pro-inflammatory cytokines, nociceptive neurotransmitters and free radicals, an increase in cytosolic calcium concentration and a general effect on the immune system. These changes produce a motor-sparing effect, because pulsed RF is selective for small unmyelinated and lightly myelinated nerve fibers (140). Contrary to conventional RF, pulsed RF should be used for a longer duration and in a repeated manner and the most common pathologies treated with pulsed RF are radicular pain, occipital and trigeminal neuralgia and shoulder and knee pain and relief can last up to several months (138, 140).

Thermal ablation reversibly ablates peripheral nerves with extreme temperatures, providing analgesia until the nerve regenerates within weeks to months, through the sprouting phenomenon (134). Besides, it also has anti-inflammatory effects and reverses oxidative stress processes (139). The magnitude of the effect on the tissue will depend on its distance to the electrode tip, the temperature reached within, the electrode size, and the procedure duration (38).

First, a LA NB is performed. If relief is obtained, then the patient is a candidate for a NB with, most commonly, bipolar radiofrequency or cryoneurolysis. When the TA is finished, a local CS may be administered to decrease post-procedure pain and possible neuritis (139).

Common indications of thermal ablation include axial pain, analgesia in failed peripheral joints, and neuroma. Although TA seems a promising treatment modality for these indications, it should be considered only when other treatment approaches are ineffective or non-practicable, and high-quality trials in chronic musculoskeletal pain are still missing.

Specific AE of thermal ablation include tendon rupture, deafferentation pain syndrome, allodynia due to aberrant neuronal regeneration, skin burns, hemorrhage, motor fiber damage and reduced motor function, hypoesthesia or dysesthesias, neuritis or neuroma formation and implant device stimulation. Pseudo-aneurysm formation and A-V fistula are both rare. RF shouldn’t be used in pregnant women, in patients with uncontrolled diabetes mellitus, or in patients with an internal defibrillator or a pacemaker with no fixed rate pacing, considering the exceptions cited above (137, 138, 140).

Specific contraindications for cryoneurolysis include Raynaud’s phenomenon, cryoglobulinemia, and cold urticaria (138).

Key take-home messages related to thermal ablation:

- TA - it uses unipolar, bipolar, pulsed, or cooled RF and cryoneurolysis.
- TA - its therapeutic action lies in the ability to modulate neural pathways responsible for pain transmission using extreme temperatures

### Intratissue percutaneous electrolysis

Intratissue Percutaneous Electrolysis (IPE) or percutaneous needle electrolysis is an USG technique that uses a galvanic current in a saline solution. It produces a non-thermal electrochemical ablation through a cathode flow, oriented toward a degenerated tendon section or to a fibrotic area. The galvanic current is delivered through a needle 0.3–0.33 mm in diameter, placed inside the target zone. IPE leads to the production of sodium hydroxide and altered pH, causing a localized inflammation and, ultimately, tissue regeneration and repair (141–143).

Most treatment protocols use a current intensity between 2 and 6 milliampere, up to 3 times per session, with a maximum of 5 s per active procedure (142).

Common indications include tendinopathies and localized fibrosis (143–147).

Specific AE are usually minor and short-lived and may include local pain or bleeding and paraesthesia (143).

The evidence for IPE effectiveness in tendinopathies is, however, scarce and influenced by small sample sizes, varying treatment protocols, clinical heterogeneity, poor trial design, and high risk of bias.

Key take-home messages related to intratissue percutaneous electrolysis:

- IPE - is a USG procedure that uses a galvanic current in a saline solution
- IPE - it produces a non-thermal electrochemical ablation delivered to the target zone, causing a localized inflammation, tissue regeneration, and repair

Table 5 shows the main differences between IPE and PENS.

**TABLE 5:** PENS and IPE: key differences.

Feature	PENS	IPE/PNE
Primary goal	Neuromodulation for analgesia	Tissue regeneration + local inflammatory stimulus
Type of current	Pulsed electrical stimulation	Direct galvanic current
Target	Peripheral nerves (sensory or motor)	Degenerative soft tissue (tendon, fascia, TrPs)
Physiological effect	Alters nerve signaling; reduces pain perception	Causes controlled micro-lysis and inflammatory cascade
Clinical use	Musculoskeletal and neuropathic pain	Tendinopathies, myofascial pain, chronic soft-tissue lesions
Sensory effect	Produces paraesthesia or muscle contraction	Produces local mechanical–chemical effect, minimal neuromodulation

PENS, percutaneous electrical nerve stimulation; IPE/PNE, intratissue percutaneous electrolysis/percutaneous needle electrolysis; TrPs, trigger-points.

### What do guidelines show?

We considered only levels of evidence (LoE) 1 and 2 (“systematic review of randomized trials” and “prospective randomized trials,” respectively), according to the guidelines from the Oxford Centre for Evidence-Based Medicine (148),^4^ used in references 151–159 and LoE 1 (“at least 1 controlled and randomized clinic trial, properly designed”), according to the guidelines from the Current Methods of the US Preventive Services Task Force (149), used in reference 73, covering different MIMSP.

The European League of Associations for Rheumatology (EULAR) guidelines for hand osteoarthritis, states that accuracy depends on the joint, route of entry, and health professional expertise and if imaging guidance is available, for example ultrasound, it may be used to improve accuracy (LOE 1–2); diabetic patients, especially those with suboptimal control, should be informed about the risk of transient increased glycaemia following IA CS and advised about the need to monitor glucose levels particularly from first to third day (LOE 1); the shared decision to reinject a joint should take into consideration benefits from previous injections and other individualized factors (e.g., treatment options, compound used, systemic treatment, comorbidities…) (LOE 2); avoid overuse of injected joints for 24 h following IAT; however, immobilization is discouraged (LOE 1) (150).

The European Federation for Ultrasound in Medicine and Biology (EFSUMB), states that US guidance should be considered for fluid aspiration, for perineural injections and to improve the accuracy of intraarticular injections of shoulders, wrists, hips, and knees and for intra-tenosynovial glucocorticoid injection in inflammatory tenosynovitis (LoE 1); USG for intra-articular injections of elbows, cervical and lumbar spines, and for sacroiliac joints might be considered as an alternative for CT or fluoroscopy guidance (LoE 2); US-guided procedures such as high-volume injection in painful Achilles chronic tendinopathy and platelet-rich plasma in plantar fasciitis, patellar tendinopathy, and epicondylitis might be considered (LoE 2); US monitoring of the needle tip should be performed throughout the injection in order to avoid intraneural needle tip placement (LoE 2); US visualization of tissue expansion/injectate spread without resultant increase of the cross-sectional area of the nerve should be sought (LoE 2) (151).

According to the European Society of Musculoskeletal Radiology (ESSR), the following statements for the shoulder region reach LoE 1: ultrasound-guided percutaneous irrigation of calcific tendinopathy (US-PICT) is a feasible, safe, and effective procedure for calcific tendinopathy treatment, both when performed with one or two needle techniques; US-PICT reduces risks of adverse events when compared to extracorporeal shockwave therapy (ESWT), US-PICT plus ESWT, and SASD corticosteroid injections; ultrasound-guided glenohumeral joint (GHJ) injections are more accurate than palpation-guided injections; ultrasound-guided and fluoroscopy-guided acromioclavicular joint (ACJ) injections are significantly more accurate than palpation-guided injections; SASD bursa injections under ultrasound guidance are feasible and tend to be more accurate than palpation-guided injections, although there is conflicting evidence about clinical superiority (152).

According to the ESSR, the following statements for the shoulder region reach LoE 2: US-PICT is more effective than simple SASD bursa steroid injection in improving symptoms and functional status of rotator cuff tendons; ultrasound-guided prolotherapy for supraspinatus tendinopathy can reduce pain and improve function better than placebo or physiotherapy; ultrasound-guided injections of steroid in the long head of biceps tendon (LHBT) sheath are more accurate and effective than palpation-guided injections; ultrasound-guided PRP injection in patients with arthroscopically repaired rotator cuff tears does not demonstrate conclusive benefit for reducing postoperative pain after arthroscopy compared to placebo; ultrasound guidance improves the outcome of GHJ injections compared to palpation-guided or sham injections in adhesive capsulitis up to 12 weeks; intra-articular ACJ local anesthetic and/or steroid injections produce pain reduction, with imaging guidance improving the outcome compared to palpation; ultrasound-guided SASD bursa injection of hyaluronic acid is more effective than placebo in patients with painful shoulder; ultrasound-guided SASD bursa corticosteroid injection is more effective than hyaluronic acid injection in patients with painful shoulder in the short term (152).

According to the ESSR, the following statement for the elbow and wrist regions is the only one reaching LoE 1: ultrasound-guided steroid wrist injections result in greater pain reduction and a greater likelihood of attaining clinically important improvement compared to palpation guidance (153).

According to the ESSR, the following statements for the elbow and wrist regions reach LoE 2: steroid injections are effective for short-term pain relief in lateral epicondylosis, but they should be avoided as a first-line treatment for chronic disease; ultrasound-guided platelet-rich plasma (PRP) injection to treat chronic lateral epicondylitis shows good results with no superiority in treatment outcomes compared to other minimally invasive procedures; ultrasound-guided steroid injection of the first extensor compartment of the wrist is safe and effective in relieving symptoms in De Quervain’s disease; in trigger finger, open surgery seems to be superior to intrasheath steroid injection but has a higher complication rate; intra-articular elbow injection under ultrasound guidance is more accurate than palpation-guided injections with greater improvement in joint function up to 6 weeks; Ultrasound guidance yields higher accuracy of joint injections around the wrist compared to palpation guidance; USG intra-articular wrist injections for inflammatory arthritis are significantly less painful than palpation guided methods and improve cost-effectiveness (153).

According to the ESSR, the following statement for the hip region is the only one reaching LoE 1: image-guided intra-articular hip injections are well-tolerated and safe procedures, which are more accurate and effective than palpation-guided injections (154).

According to the ESSR, the following statements for the hip region reach LoE 2: image and palpation-guided corticosteroid-anesthetic injections are both feasible, safe, and effective to treat greater trochanteric pain syndrome (GTPS), providing clinical improvement up to 3–6 months, but USG injections seem to be more effective, compared to palpation-guided injections, when performed into the greater trochanteric bursa; USG CS injection, needling, and PRP injection for GTPS are all valuable measures to reduce pain and no clear evidence exists to define one treatment as superior to the others, but PRP may have more long-lasting clinical improvement than CS injections, although high-quality evidence is still missing; image-guided corticosteroid hip injection is effective in providing short-term pain relief and can transiently improve function in patients with osteoarthritis; USG intra-articular hip injection of hyaluronic acid is not different from placebo for pain and function, nor from CS (at 1 and 6 months), or PRP (at 6 and 12 months) in patients with hip osteoarthritis and no different outcomes have been observed by using different hyaluronic acid formulations (154).

According to the ESSR, the following statements for the knee region reach LoE 1: intra-articular USG procedures around the knee joint, such as arthrocentesis and intra-articular injections, are more accurate than palpation-guided procedures, resulting in improved fluid aspiration and injection therapeutic outcome(s); USG dry needling is effective in improving function and pain in patellar tendinopathy, especially if associated with PRP, although conflicting results about the clinical effectiveness of PRP in patellar tendinopathy do not allow supporting the use of this treatment as a first line approach (155).

According to the ESSR, the following statement for the knee region reaches LoE 2: USG knee joint injections of corticosteroid/anesthetic give short-to-midterm pain relief and functional improvement in inflammatory arthritis and although similar outcomes may be observed in osteoarthritis, efficacy is controversial, and alternative analgesic therapies (such as oxygen-ozone) have been proposed, but evidence supporting their use remains limited (155).

According to the ESSR, the following statements for the ankle and foot regions reach LoE 1: Image-guided injections are safe and feasible to treat Achilles degenerative tendinopathy, but there is insufficient evidence from randomized controlled trials (RCT) to support them over conservative therapies; US-guided PRP injections for plantar fasciitis are safe and provide significant pain relief in chronic plantar fasciitis, with better clinical outcome at mid- and long-term follow-up if compared with CS injections; USG CS injections are the most effective image-guided interventional procedure to improve pain in patients with Morton’s neuroma, especially in the first 3 months; USG improves the effectiveness of different interventional procedures for Morton’s neuroma if compared to palpation guidance, particularly for CS injection (156).

According to the ESSR, the following statements for the ankle and foot regions reach LoE 2: USG CS injections are more effective than palpation-guided injections to treat plantar fasciitis providing significant short-term pain relief, particularly when combined with strength training and stretching; USG ethanol injection of Morton’s neuroma is relatively safe and well-tolerated, but further investigations are required to clearly demonstrate its clinical value before supporting this procedure (156).

According to the ESSR, the following statements for the upper limb nerves reach LoE 1: ultrasound guidance is a safe and effective method for brachial plexus block; USG non-surgical approaches are safe and effective methods to treat carpal tunnel syndrome in the short term, but there is sparse evidence in the mid- and long-term effectiveness of these interventions (157).

According to the ESSR, the following statements for the upper limb nerves reach LoE 2: USG pulsed radiofrequency ablation of the suprascapular nerve for adhesive capsulitis combined with physical therapy provides good clinical outcome at 12 weeks follow-up; USG steroid injection is feasible in patients with ulnar neuropathy at the elbow but is not superior to placebo; USG perineural circumferential hydrodissection is a feasible procedure to treat median nerve entrapment (157).

According to the ESSR, the following statements for the lower limb nerves reach LoE 2: image-guided pudendal nerve block is safe, effective, and well tolerated with few complications; USG perisciatic injection of anesthetic provides good symptom relief in patients with piriformis syndrome, but the addition of CS to local anesthetics still has an unclear role; USG lateral femoral cutaneous nerve block can be used to provide effective post-operative regional analgesia and the volume of local anesthetic affects the size of the blocked sensory area (158).

According to D’Souza et al. (72), the following statements reach LoE 1: PRP injection for lateral epicondylosis is associated with superior long-term relief (3 months to 2 years) compared to CS injection; intra-tendinous ACP injection is likely ineffective for improving pain control or functionality in lateral epicondylosis and other tendinopathies; there is inconsistent and conflicting evidence on the use of PRP and BMAC for treatment of Achilles tendinopathy and the re-rupture rate was not different with PRP injection versus saline injection; there is low-quality evidence supporting superiority of dextrose prolotherapy compared to placebo for plantar fasciitis, although moderate-quality evidence suggests that it is inferior to CS injections and therefore, prolotherapy is not the first recommended injection option for plantar fasciitis; there is consistent evidence supporting that PRP injections for plantar fasciitis are associated with superior analgesia and physical function compared to placebo and CS injections over the long-term (6–24 months), although outcomes may be comparable in the short-term; the use of PRP injection for rotator cuff tendinosis is likely to provide superior analgesia compared to corticosteroid injections, including intra-articular, subacromial, and intra-tendinous approaches, although studies with non-inferior outcomes utilized less than two times the platelet concentrations, highlighting the importance of having a minimum concentration of four to five times in the PRP injectate; there is inconsistent but promising evidence from a few RCTs on the use of PRP for hamstring and gluteal medius/minimus tendinopathy; there is limited evidence to suggest that a strategy of PRP with physical therapy is superior to a strategy with just physical therapy alone for treatment of muscle injuries and current evidence suggests there is likely minimal to no benefit; PRP injection for knee OA is associated with superior long-term analgesia and physical functioning outcomes compared to HA, CS injection, and placebo at 6 and 12 months; there is inconsistent and conflicting evidence on the use of MSCs to improve pain intensity and physical function in patients with knee OA and although some RCTs have indicated superior analgesia, functional ability, walking distance, and quality of life with MSC injection, other studies have failed to replicate this outcome; although intra-articular PRP injection may lead to improvements in analgesia and physical function in patients with hip OA, there may be no difference when compared to other interventions like HA injections; there is consistent evidence, albeit from a few RCTs, that PRP injection for shoulder OA is associated with long-term improvement in pain and function compared to corticosteroid injection; there is inconsistent and limited evidence regarding the use of PRP and BMAC for osteochondral lesions or OA of the foot or hand and current evidence does not support the routine use of injectable biologics for this indication compared to conventional therapy; PRP injection in the lumbar facet joints may be associated with superior analgesia, physical function, and patient satisfaction compared to local anesthetic and corticosteroid injection for lumbar facet- mediated pain; the current evidence generally supports that intra-articular sacroiliac joint injection with PRP may be associated with superior analgesia compared to intra-articular CS injection for sacroiliac joint pain; the current evidence generally suggests that intra-discal PRP injection may lead to improvement of discogenic pain (or pain from intervertebral disc disease) compared to placebo, CS injections, HA, and other injections, but there is substantial clinical heterogeneity among studies, especially with respect to platelet concentration as well as LP-PRP versus LR-PRP formulations with non-superiority of PRP treatments in studies using low-volume leukocyte-poor injectate; the current evidence suggests that intra-discal BMAC injection may provide long-term alleviation of pain and improvement in physical function for patients with discogenic pain, although these differences may be similar to those with intra-discal injection with PRP; very limited evidence suggests that perineural injection of PRP along certain nerves (e.g., median, ulnar, radial, peroneal, tibial, saphenous, and/or sural) may be associated with improvements in pain intensity and neurological symptoms (e.g., numbness) in patients with diabetic peripheral neuropathy compared to conventional medical management; limited and conflicting evidence suggests that intrathecal or in-lesion injection with MSCs for spinal cord injury may decrease chronic pain intensity, but because of inconsistent and limited data, the current evidence does not support the routine use of intrathecal or in-lesion biologic injection for spinal cord injury; while epidural injection with ACS may improve radicular pain symptoms, some evidence suggests that it is not superior to CS injection; epidural injection with PRP or other PRP-related products (e.g., PL) may alleviate radicular pain symptoms in radiculopathy, although studies had a high risk for bias; autologous fat graft injection at the site of pain for post-herpetic neuralgia is not associated with superior analgesia compared to saline/placebo injection; comparative efficacy between injectable biologics, such as PRP versus MSCs, for various clinical indications (e.g., knee OA) is likely of insignificant difference, although direct comparative studies suggest that PRP and MSC show no difference in analgesic and physical functioning outcomes; injectable biologics, notably PRP and MSCs, have been shown to be a safe treatment modality with minimal adverse effects related to the injection (localized soreness, bruising, infection, bleeding) and severe adverse reactions are very rare and may consist of neoplasm formation, disease transmission, reactivation of latent viruses, and graft-versus-host disease (72).

The osteoarthritis guidelines concerning the intra-articular treatment from the Osteoarthritis Research Society International (OARSI), the American College of Rheumatology (ACR), the European Society for Clinical and Economic Aspects of Osteoporosis, Osteoarthritis and Musculoskeletal Diseases (ESCEO) and the Department of Veterans Affairs/Department of Defense (VA/DoD), follow the Grading of Recommendations Assessments, Development and Evaluation (GRADE) methodology (159).^5^ The osteoarthritis guidelines concerning the intra-articular treatment from the National Institute for Health and Care Excellence (NICE) and from the American Academy of Orthopedic Surgeons (AAOS) follow recommendations of their own (160, 161). ^6^

The OARSI guidelines recommend conditionally intra-articular CS and intra-articular hyaluronic acid in knee osteoarthritis, not in hip osteoarthritis, nor in polyarticular osteoarthritis (162).

The ACR guidelines strongly recommend intra-articular CS for hip and knee OA and conditionally recommend radiofrequency ablation for knee OA and intra-articular CS for hand OA. These guidelines are conditionally against hyaluronic acid for hand and knee OA, and botulinum toxin and prolotherapy for knee or hip OA. They are strongly against PRP and MSC for hip or knee OA and strongly against hyaluronic acid for hip OA (163).

The EULAR guidelines for hand OA state that intra-articular corticosteroids should not generally be used in patients with hand OA, but may be considered in patients with painful interphalangeal joints. These guidelines are strongly against prolotherapy, mesenchymal stem cells, nerve blocks, and PRP (164).

The ESCEO guidelines for knee OA afford a weak recommendation for intra-articular CS, and for hyaluronic acid in patients who are still symptomatic despite the use of NSAIDs or have a contraindication to NSAIDs (165).

The VA/DoD guidelines afford a weak recommendation for intra-articular CS or hyaluronic acid injections in knee OA and for guided intra-articular CS injection in hip OA, in patients with persistent pain due to OA, inadequately relieved by other interventions. These guidelines afford a weak recommendation against the use of intra-articular hyaluronic acid injection of the hip, being neither for nor against the use of PRP injections for the treatment of knee or hip OA, due to insufficient evidence (166).^7^

The AAOS guidelines state that there is strong evidence against the use of intra-articular hyaluronic acid in the treatment of gleno-humeral joint OA, and by consensus, there is an absence of reliable evidence supporting the use of injectable biologics such as stem cells or PRP, which cannot be recommended in the treatment of the same condition (161). The AAOS affords a moderate recommendation for intra-articular CS to improve function and reduce pain in the short-term for patients with symptomatic hip OA and affords a strong recommendation against the use of intra-articular hyaluronic acid in the treatment of the same condition (167).^8^ It also affords a moderate recommendation against the routine use of intra-articular hyaluronic acid and a moderate recommendation in favor of intra-articular CS in the treatment of patients with symptomatic knee OA, considering that these can benefit from the short-term relief provided by CS (168).^9^ The AAOS guidelines also afford a limited recommendation in favor of PRP and denervation therapy (thermal or chemical), to reduce pain and improve function in patients with symptomatic knee OA, and state by consensus that the utility/efficacy of dry needling requires additional evidence in the same population (168).

The NICE guidelines recommend not to offer intra-articular hyaluronic acid to manage OA, but that intra-articular CS injections may be considered when other pharmacological treatments are ineffective or unsuitable, or to support therapeutic exercise, explaining to the patient that these only provide short-term relief (161).

## Conclusion

US guidance ensures proper needle positioning, increases efficacy and tolerance, reduces adverse events, and is more cost-effective than landmark or fluoroscopic-guided procedures.

MIMSPs are used for both diagnostic and therapeutic purposes in RMD, and a considerable variety of techniques are available for treating soft tissue and articular pathologies.

Intra-articular and soft tissue drug delivery has several advantages over systemic delivery, namely reduced systemic exposure and adverse events, increased local bioavailability, and even lower costs. However, the efficacy of some of the injectables used in intra-articular and soft tissue therapies remains controversial due to a lack of data, methodological limitations in low-quality studies, heterogeneity in studied populations and in study design, and absence of high-level evidence. Moreover, patient-reported outcomes such as pain and stiffness are responsive to the placebo effect, which is particularly significant when injectables are used. Other factors, like non-standardization in drug delivery and residence time, also pose problems, particularly in cell therapies, when attempting to ascribe clinical meaning to therapy interventions. Finally, clinical guidelines regarding MIMSP are sometimes inconsistent with one another.

## Footnotes

1
http://nysora.com/techniques-archive

2
http://acep.org/sonoguide/nerve-blocks

3
https://pain.ucsf.edu/topics/interventional-pain-management

4 OCEBM Levels of Evidence working Group (2011). The Oxford Levels of Evidence 2. Oxford Centre for Evidence-Based Medicine (https://www.cebm.ox.ac.uk/resources/levels-of-evidence/ocebm-levels-of-evidence).

5
https://www.gradepro.org

6
https://www.nice.org.uk/guidance/ng226/chapter/Recommendations#pharmacological

7
https://www.healthquality.va.gov/HEALTHQUALITY/guidelines/CD/OA/VADODOACPG.pdf

8
https://www.aaos.org/globalassets/quality-and-practice-resources/osteoarthritis-of-the-hip/oah-cpg.pdf

9
https://www.aaos.org/quality/quality-programs/osteoarthritis-of-the-knee
